# Glory of Indian railways

**DOI:** 10.1136/jech-2020-214734

**Published:** 2020-06-30

**Authors:** Soumya Chippagiri

**Affiliations:** Department of Community Health, St. John’s Medical College, Bangalore, India

**Keywords:** PREVENTIVE MEDICINE, PRIMARY healthcare, PRIMARY CARE, PUBLIC HEALTH, PREVENTION

**Figure 1:**
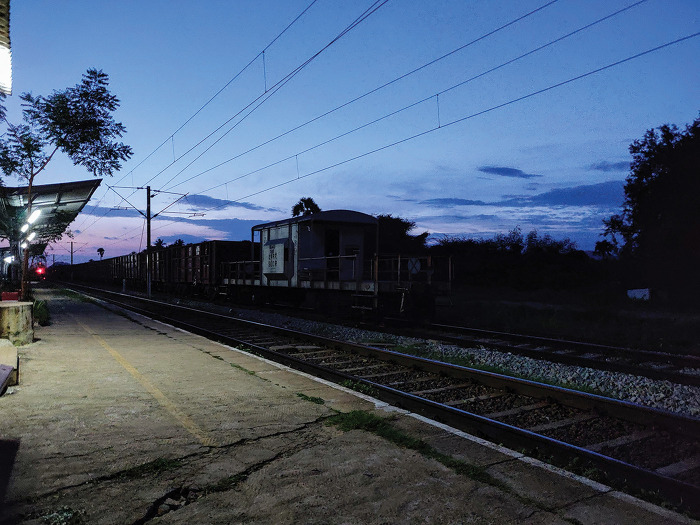
A train to battle pandemic.

Indian railways are known to be one of the largest in the world. So much so that it has two UNESCO World Heritage Sites. It was the pandemic, coronavirus disease 2019 (COVID-19) that brought this lifeline to a standstill in 167 years. India being the resilient and innovative country that it is, has left no stone unturned to curtail the spread and control the infection. The trains are converted to COVID wards. Each coach is well armed to accommodate at least 16 patients, one doctors and nursing station each, space for medical equipment and facility for sanitation. This coach is enabled to navigate to the deepest parts of the country in case of a spike in COVID-19 cases when the hospitals are overwhelmed. Acting as a mobile ward, it transport patients when necessary from rural to the healthcare-rich urban areas. Indian trains are being used in a healthful manner since 1991 with the Lifeline Express or Jeevan Rekha Express.

